# Cardiovascular and Hematological Risk Factors and Mortality Risk in Pediatric Arterial Ischemic Stroke: Analysis Report From Hospitals in the United States

**DOI:** 10.7759/cureus.10859

**Published:** 2020-10-09

**Authors:** Nitya Beriwal, Hira Imran, Edmond Okotcha, Kosisochukwu Oraka, Saurabh Kataria, Renu Bhandari, Rikinkumar S Patel

**Affiliations:** 1 Medicine, Lady Hardinge Medical College, New Delhi, IND; 2 Medicine, Rawalpindi Medical University, Rawalpindi, PAK; 3 Medicine, Vinnytsia National Medical University. N. I. Pirogov, Vinnytsia, UKR; 4 Neurology and Neurocritical Care, University of Missouri Health Care, Columbia, USA; 5 Neurology, West Virginia University, Morgantown, USA; 6 Medicine, Manipal College of Medical Sciences, Kaski, NPL; 7 Psychiatry, Griffin Memorial Hospital, Norman, USA

**Keywords:** ischemic stroke, pediatric stroke, pediatric mortality, medical comorbidities

## Abstract

Objective

We aimed to analyze the differences in demographics, comorbidities, and the risk of in-hospital mortality in pediatric arterial ischemic stroke (PAIS) inpatient population by hematological (HEM) and cardiovascular (CV) risk factors.

Methods

A total of 4,036 inpatients (1-18 years of age) from the Nationwide Inpatient Sample (NIS) with a primary diagnosis of PAIS were included. Descriptive statistics, linear-by-linear association test, and logistic regression models were utilized to analyze differences in demographics, comorbidities, and their impact on mortality in PAIS inpatients by CV and HEM risk factors.

Results

The cumulative in-hospital mortality rate in the entire PAIS inpatient cohort was 3.6%. The mortality rate was higher in the CV cohort (57.4%) as compared to the HEM cohort (29.7%). When compared with the cohort with no risk factors, HEM and CV were associated with four times (95% CI: 2.36-8.03) and seven (95% CI: 4.03-12.61) times higher odds for in-hospital mortality respectively. CV risk factors like cardiomyopathy and diabetes, and HEM risk factors like blood disorders, coagulation disorders, and deficiency anemias were associated with a significantly increased risk of in-hospital mortality.

Conclusion

The in-hospital mortality risk in PAIS patients was increased by 613% by CV risk factors and by 336% by HEM risk factors. Early identification and effective management of associated CV and HEM risk factors in the PAIS patient population can pave the way for increased survival and improved clinical outcomes.

## Introduction

Stroke is one of the 10 leading causes of mortality in children and adolescents, with morbidity aggravated by the long-term motor and sensory deficits, and cognitive and social deficits [1]. Pediatric ischemic stroke (PIS) includes both pediatric arterial ischemic stroke (PAIS) and cerebral sino-venous thrombosis (CSVT). PAIS alone accounts for 80% of the perinatal strokes with PIS having an incidence of one in 3,500 to 10,000 newborns, and the incidence of PIS in children (non-neonates) is one to two in 100,000 children annually in developed countries in the west [2]. The incidence of PAIS and CSVT is 2.6 and 0.7 in 100,000 children per year respectively [3,4]. This suggests that PAIS is a clinically predominant entity, as the majority of PIS cases in children are accounted for by PAIS.

An increase of around 35% was seen in the absolute PIS cases from 1990 to 2013 [5]. It is a public health concern due to the associated increase in healthcare burden, with a first-year post-stroke cost of $15,000 to $140,000 per PIS hospitalization. This calculation underestimates the actual financial burden on the family as it is compounded by the effect of long-term neurological sequelae and disability on social functionality for the child and family [1].

Risk factors commonly associated with PAIS include arteriopathies, cardiovascular (CV) diseases (cardiomyopathies, congenital heart diseases), hematological (HEM) disorders (prothrombotic states, sickle cell disease, HEM malignancy, deficiency anemias), acute systemic illnesses (fever, dehydration, sepsis, infection), chronic systemic illnesses [systemic lupus erythematosus (SLE), connective tissue disorders], and risk factors predisposing to atherosclerosis in adulthood (hypertension, hyperlipidemia, diabetes mellitus) [4,6,7]. As per data from the International Pediatric Stroke Study, arteriopathies (53%) and CV disorders (31%) were the most common risk factors in PAIS [7].

Mortality rates of 10.5% were seen with PIS in a nationwide population-based study enrolling 685 pediatric stroke cases with 36.1% PIS cases in Taiwan [8]. According to a Kids' Inpatient Database study, PIS has a hospitalization rate of 3.7 per 100,000 children, with the highest rates seen in children under four years of age and those between 15-20 years of age [9]. Mortality-based risk factor stratification is essential for early recognition of at-risk patients for appropriate clinical management to reduce long-term disease burden and fatality. Mortality risk of treatable or modifiable risk factors needs to be studied. In our study, we aim to assess the differences in demographics and comorbidities in PAIS inpatients by CV versus HEM risk factors. Next, we aim to assess the risk of in-hospital mortality due to HEM risk factors after controlling for demographics and CV risk factors.

## Materials and methods

We included inpatients (aged 1-18 years) from the Nationwide Inpatient Sample (NIS, 2010-2014) in our cross-sectional study. The NIS provides clinical information coded using the International Classification of Diseases, Ninth Edition (ICD-9), and Clinical Classifications Software (CCS) codes. The data were obtained from 4,400 hospitals across 45 states in the United States. As the patients' identities and their health-related information were de-identified, we did not require institutional review board approval [10].

A total of 4,036 pediatric inpatients with a primary discharge diagnosis of acute arterial ischemic stroke, i.e., PAIS were included using ICD-9 codes 433.01, 433.11, 433.21, 433.31, 433.81, 433.91, 434.01, 434.11, 434.91, or 436. The study inpatients were further grouped by comorbid CV risk factors (N = 1,321) including congenital heart anomalies, cardiomyopathy (pericarditis, myocarditis, endocarditis, hypertrophic cardiomyopathy, metabolic cardiomyopathy, cardiac tamponade, and rheumatic heart disease), diabetes, hypertension (complicated and uncomplicated), and obesity, and HEM risk factors (N = 1,161) including connective disorders (SLE, polymyalgia rheumatica, systemic sclerosis, sicca syndrome, dermatomyositis, and polymyositis), sickle cell anemia, coagulation disorders (acquired coagulation factor deficiency, thrombocytopenia, Von Willebrand disease, and coagulation factors viii, ix, and xi deficiency), deficiency anemias (pernicious anemia, folate deficiency anemia, megaloblastic anemia, nutritional anemia, sideroblastic anemia, anemia of chronic disease, and iron deficiency anemia), and blood disorders (lymphoma, leukemia, and multiple myeloma). These comorbid risk factors were selected based on current literature [6,7].

Demographic variables studied included age (1-18 years), sex (male and female), and race (White, Black, Hispanic, and others). We measured the in-hospital mortality between subgroup cohorts, and in the NIS, in-hospital mortality is described as all-cause [11].

We used cross-tabulation and descriptive statistics to discern the differences in demographics and comorbidities in PAIS inpatients by CV versus HEM versus no risk factors. A linear-by-linear association test was used to assess the significance across the cohorts. Logistic regression models were used to evaluate the impact of HEM and CV risk factors on the odds ratio (OR) association with in-hospital mortality after controlling the model for demographic confounders. Another regression model was used to evaluate a detailed risk factor stratification by comorbidities with in-hospital mortality. A p-value of <0.05 determines the statistical significance in data analysis conducted using the SPSS Statistics version 26 (IBM Corporation, Armonk, NY).

## Results

The mean age of PAIS inpatients with CV risk factors (9.9 ±6.3 years) was higher compared to the rest of the inpatients. The majority of the inpatients with HEM risk factors were children <11 years of age (63.9%), and those with CV risk factors were mainly adolescents (12-18 years, 48.2%). HEM risk factors were more prevalent in females (51.4% vs. 48.6% in males, p: <0.001), and CV risk factors were seen in a higher proportion in males (52% vs. 48% in females, p: <0.001). While PAIS inpatients with CV risk factors were predominantly Whites (52.8%), inpatients with HEM risk factors were predominantly Blacks (38.8%), and there was a statistically significant difference race-wise among PAIS inpatients by risk factors. In-hospital mortality was seen in a higher proportion in PAIS inpatients with CV risk factors (57.4%) compared to inpatients with HEM risk factors (29.7%), as shown in Table 1.

**Table 1:** Demographic distribution and in-hospital mortality in pediatric arterial ischemic stroke inpatients SD: standard deviation

Variable	No risk factors	Cardiovascular risk factors	Hematological risk factors	P-value
Total inpatients	1,554	1,321	1,161	-
Mean age, years (SD)	8.9 (6.32)	9.9 (6.34)	8.4 (5.97)	<0.001
Age at admission, %				
1-5 years	35.3	30.0	40.0	0.012
6-11 years	23.1	21.8	23.9	
12-18 years	41.6	48.2	36.1	
Sex, %				
Male	56.0	52.0	48.6	<0.001
Female	44.0	48.0	51.4	
Race, %				
White	57.8	52.8	36.8	0.001
Black	13.1	20.5	38.8	
Hispanic	19.5	16.9	13.3	
Others	9.6	9.8	11.1	
In-hospital mortality, %	12.8	57.4	29.7	<0.001

**Figure 1:**
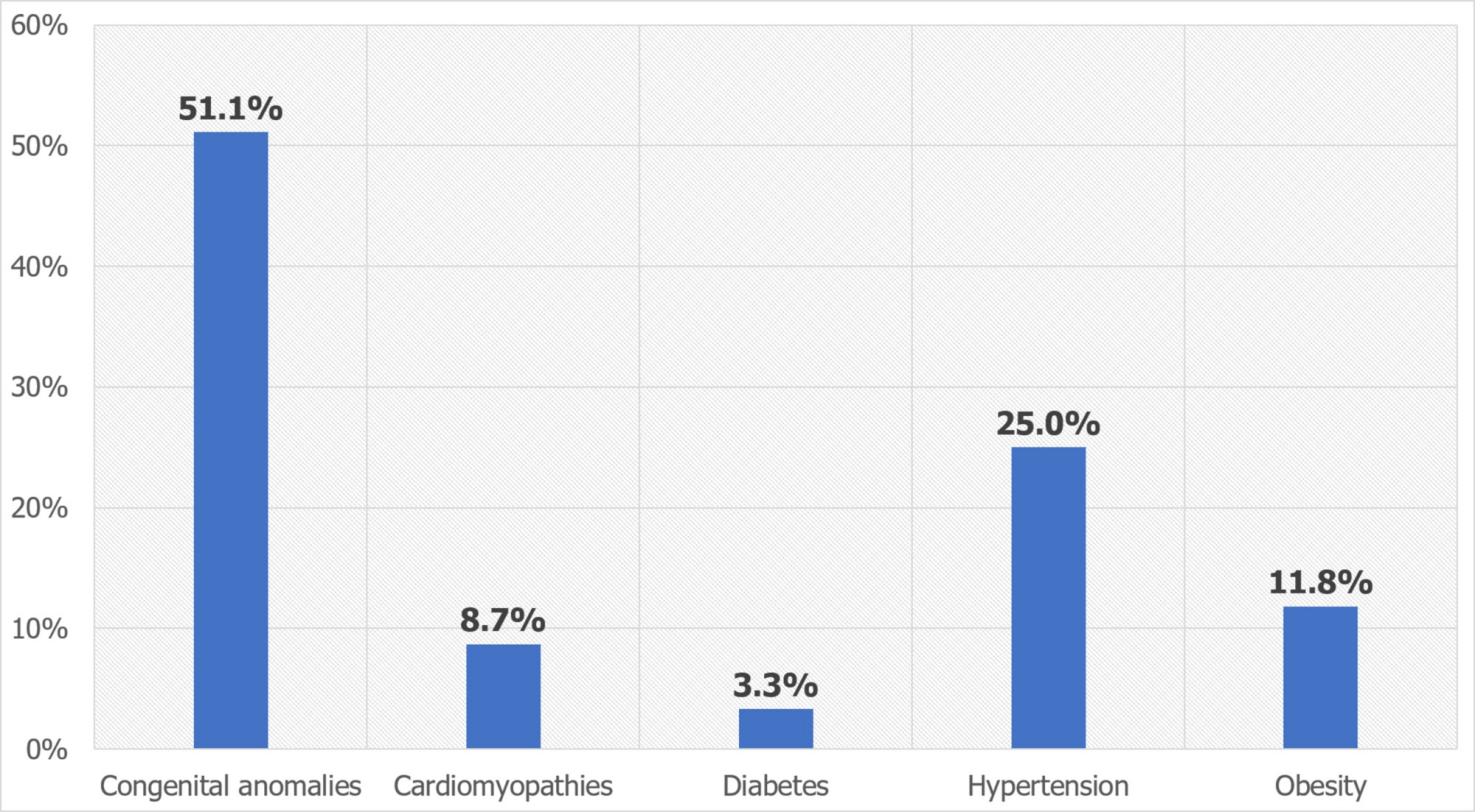
Distribution of cardiovascular risk factors in pediatric arterial ischemic stroke inpatients

**Figure 2:**
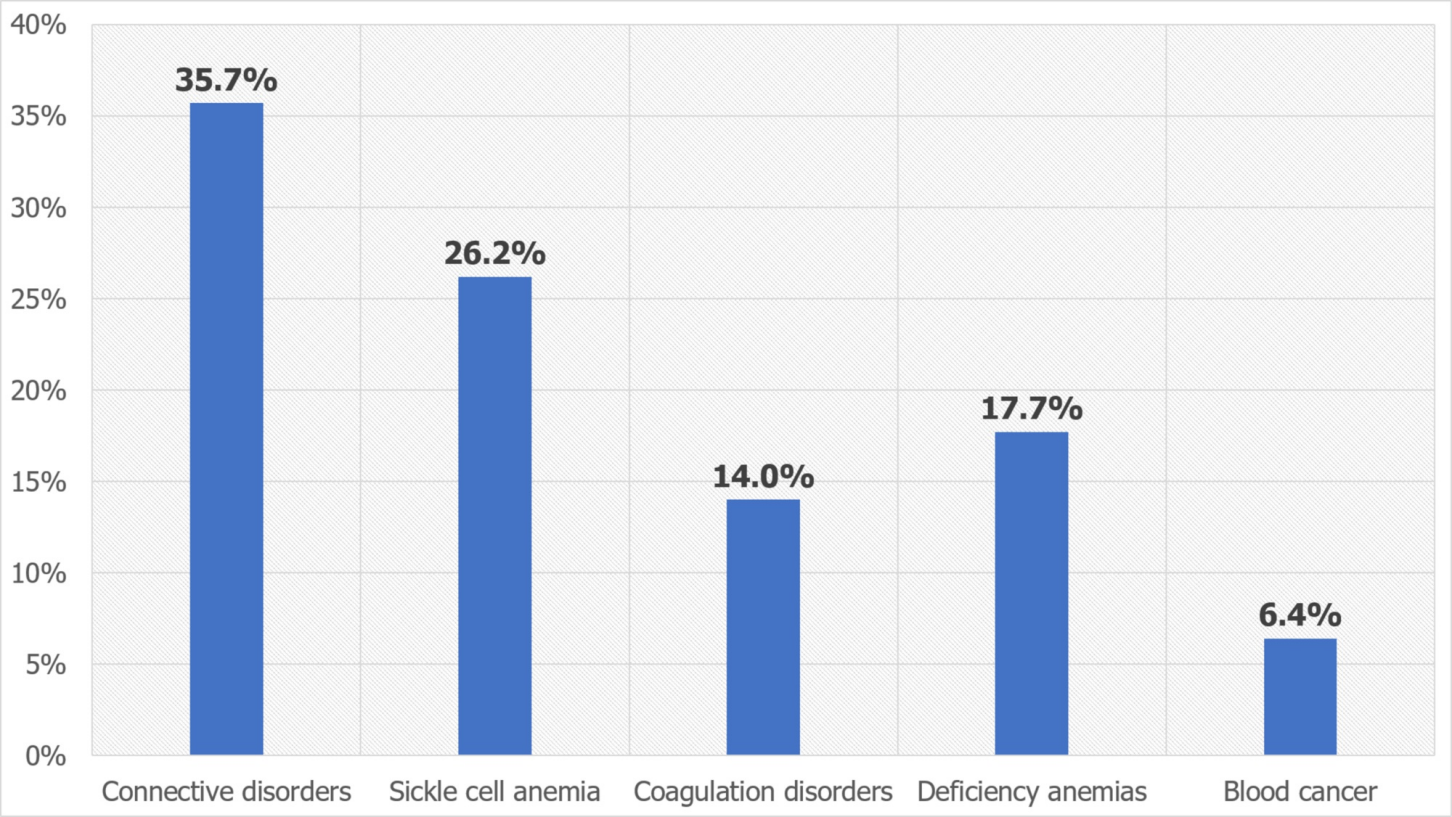
Distribution of hematological risk factors in pediatric arterial ischemic stroke inpatients

The most common CV risk factors in PAIS inpatients were congenital anomalies (51.1%), hypertension (25%), and obesity (11.8%), whereas cardiomyopathy (8.7%) and diabetes (3.3%) were less prevalent comorbidities. On the other hand, the most common HEM risk factors were SLE/connective tissue disorders (35.7%), sickle cell anemia (26.2%), deficiency anemias (17.7%), and coagulation disorders (14%), whereas blood cancers including lymphoma, leukemia, and multiple myeloma were less prevalent (6.4%), as shown in Figures 1, 2.

Children under the age of 11 years had higher odds for in-hospital mortality, with children in the age group of one to five years having 1.68 times (95% CI: 1.18-2.54) and those in the age group of 6-11 years having 1.74 times (95% CI: 1.11-2.72) higher odds. Demographic characteristics like sex and race were statistically not significant predictors for in-hospital mortality.

The mortality rate seen in the entire PAIS inpatient cohort was 3.6% (148 inpatient deaths out of 4,036), and about 3.1% was accounted for by inpatients with HEM and CV risk factors. When compared with inpatients with no risk factors, those with HEM and CV risk factors had 4.4 times (95% CI: 2.36-8.03) and 7.1 times (95% CI: 4.03-12.61) higher odds for in-hospital mortality respectively, as shown in Table 2.

**Table 2:** Predictors of in-hospital mortality in pediatric arterial ischemic stroke inpatients

Variable	Logistic regression model			
	Odds ratio	95% confidence interval		P-value
		Lower	Upper	
Age at admission				
1-5 years	1.68	1.18	2.54	0.013
6-11 years	1.74	1.11	2.72	0.016
12-18 years	Reference			
Sex				
Female	1.14	0.80	1.61	0.470
Male	Reference			
Race				
White	Reference			
Black	0.64	0.41	1.01	0.057
Hispanic	0.77	0.46	1.28	0.317
Other	0.90	0.51	1.60	0.720
Risk factors				
None	Reference			
Cardiovascular	7.13	4.03	12.62	<0.001
Hematological	4.36	2.37	8.03	<0.001

We then conducted another logistic regression analysis adjusted for demographic confounders and found that among CV risk factors, cardiomyopathies and diabetes were potential comorbidities that increased the odds of in-hospital mortality by 15.6 times (95% CI: 9.19-26.56), and 11.2 times (95% CI: 5.01-24.86) respectively. Among PAIS inpatients with HEM risk factors, blood cancer was associated with 4.8 times (95% CI: 2.236-10.086), coagulation disorders with 2.2 times (95% CI: 1.277-3.898), deficiency anemias with 1.9 times (95% CI: 1.130-3.339) higher risk of in-hospital mortality. Both connective tissue disorders and congenital anomalies were the most prevalent risk factors in PAIS inpatients but did not increase the risk of mortality. Sickle cell anemia and congenital abnormalities had a statistically non-significant mortality risk, as shown in Table 3.

**Table 3:** In-hospital mortality risk in pediatric arterial ischemic stroke inpatients stratified by risk factors

Variable	Logistic regression model			
	Odds ratio	95% confidence interval		P-value
		Lower	Upper	
Cardiovascular risk factors				
None	Reference			
Congenital anomalies	1.38	0.79	2.38	0.253
Cardiomyopathies	15.62	9.19	26.56	<0.001
Diabetes	11.16	5.01	24.86	<0.001
Hypertension	2.39	1.31	4.37	0.005
Obesity	2.34	0.91	6.00	0.077
Hematological risk factors				
None	Reference			
Connective tissue disorders	0.28	0.11	0.70	0.007
Sickle cell anemia	1.39	0.62	3.12	0.417
Coagulation disorders	2.23	1.28	3.89	0.005
Deficiency anemias	1.94	1.13	3.34	0.016
Blood cancer	4.75	2.24	10.09	<0.001

## Discussion

Our study found that CV risk factors of cardiomyopathies and diabetes and HEM risk factors of blood cancer, coagulation disorders, and deficiency anemias were associated with a higher risk of in-hospital mortality.

About two-fifths of the patients with PAIS were adolescents (42.2%), followed by children aged one to five years (34.9%). Children under 11 years predominantly had HEM risk factors, and adolescents mainly had CV risk factors. This could be attributed to the fact that atherosclerosis-related risk factors such as hypertension, diabetes, and obesity are predominantly seen in children above the age of 12 years; 52.6% of our cohort was boys, which is suggestive of the male predominance in the PAIS inpatient population. HEM risk factors were more common in females and Blacks, whereas CV risk factors were predominantly found in males and Whites. Our findings are further supported by past studies that showed a male predominance in PAIS, and no significant association was seen between sex and mortality [7,12]. In a small prospective study (N = 96) by Mallick et al., infants and Asian and Black children were associated with a higher risk of PAIS [13]. In our study, children with PAIS in the age group of one to five years had 68% and those in the age group of 6-11 years had 74% times higher risk of in-hospital mortality. However, no significant association with inpatient mortality was observed with demographic parameters like sex and race.

The most common CV risk factors seen in PAIS inpatients included congenital anomalies, hypertension, and obesity. The predominant HEM factors were SLE/connective tissue disorders, sickle cell anemia, deficiency anemias, and coagulation disorders. These findings are supported by a study based on the Canadian Inpatient Stroke Registry data, which reported that arteriopathy (49%), cardiac disorders (28%), and prothrombotic disorders (35%) are the predominant risk factors for PAIS [4]. The International Pediatric Stroke Study enrolled 676 children between 29 days to 18 years of age and found that CV and HEM risk factors were associated with PAIS, including arteriopathies (53%), cardiac diseases (31%), prothrombotic conditions (13%), chronic systemic conditions [connective tissue disorders, sickle cell anemia, and hematological malignancies (19%)] [7]. The prevalence of cardiac disorders was highest in younger children (three to five years of age), and arteriopathies and chronic systemic conditions were mainly seen in children aged five to nine years. Also, prothrombotic states were predominantly seen in children aged 10-14 years and 15-18 years. This supports our findings on the importance of HEM and CV risk factors in PAIS.

About 57.4% of PAIS patients with CV risk factors died and had 613% times increased risk of in-hospital mortality compared to those without CV risk factors. On the contrary, about one-third of the PAIS patients with HEM risk factors died and had a 336% higher risk of mortality. In literature, mortality related to PAIS has been seen to vary from 7 to 28% [1]. However, this generally includes both in-hospital and long-term follow-up mortality. As per the Canadian Pediatric Ischemic Stroke Registry data (N = 1,129), a stroke-specific mortality rate of 5% was observed in children with PAIS [4]. A mortality rate of 10.5% was seen in PIS patients in a nationwide population-based study in Taiwan [8]. Simonetti et al.'s study based on the Swiss NeuroPaediatric Stroke Registry (N = 116) found long-term mortality of 14% in children with PAIS [14]. On the contrary, in a single-center prospective study (N = 119), the 30-day mortality in PAIS was found to be 11.7% [15]. In a study by Beslow et al. enrolling 915 neonates and 2,273 children with PAIS based on a multinational stroke registry, a mortality rate of 1.5% in neonates, 3.1% in children (29 days to <19 years of age), and a cumulative mortality rate of 2.6% were seen [16]. Their findings are mostly consistent with our study findings of an overall 3.6% in-hospital mortality rate in children aged 1-18 years.

Our study indicates that cardiomyopathies and diabetes are potential CV risk factors in patients with PAIS that increase the risk of in-hospital mortality by 1,462% times and 1,016% times respectively. Among HEM risk factors, blood cancer was associated with 374%, and coagulation disorders with 123%, and deficiency anemias with 94% higher risk of in-hospital mortality in PAIS. In a study by Chung et al., HEM risk factors (including thalassemia, aplastic anemia, hemophilia, and leukemia) and hemorrhagic transformation of PAIS and were associated significantly with high mortality rate. This supports the high mortality risk seen with HEM risk factors in our study [17]. A study by Lanthier et al. has shown that multiple risk factors predispose to risk for PIS recurrence associated with a higher mortality rate. Therefore, even in a patient with a known etiology, a complete workup including cardiovascular evaluation (angiography), hematologic evaluation, and metabolic studies must be done to rule out other risk factors that can contribute to a significant mortality risk [18].

Lopez-Espejo et al. have stated that the risk of in-hospital mortality in PAIS was significantly associated with pediatric National Institutes of Health Stroke Scale (NIHSS) score, a measure of stroke severity, and follow-up mortality risk factors included congenital heart diseases and prothrombotic states. Patients having both anterior and posterior circulation stroke, and those with prothrombotic states had a significant association with long-term mortality risk on multivariate analysis. Furthermore, they observed a statistically significant association of neoplasms with mortality on univariate Cox regression analysis, which supports our findings of a significant association of blood cancer with in-hospital mortality [15]. In our study, congenital heart anomalies were the most prevalent risk factors in PAIS inpatients but had a non-significant association with in-hospital mortality risk. There is mixed evidence in this regard, Lopez-Espejo et al. did not find a statistically significant correlation of congenital heart diseases with in-hospital mortality, which is consistent with our findings; however, the study by Beslow et al. noted a significant association of congenital heart diseases with in-hospital mortality in PAIS [15,16].

There are some limitations to our study. We used the NIS database, thereby conducting a cross-sectional study that could not find a causal relationship between all-cause mortality and associated risk factors. As per the study by Golomb et al., it has been suggested that the usage of ICD-9 codes has been shown to miss out PAIS patients as they sometimes may be assigned incorrectly [19]. We did not include infants and hence did not take perinatal strokes and PAIS in infants into consideration. Also, we did not take into account CSVT, which also contributes to PIS; however, PAIS has a higher incidence in the pediatric population. Regardless of these potential limitations, the NIS database offers an incomparable population-based perception of disease associations with systematic and temporal factors, which was previously used in an adult stroke study. Its data provides a foundational basis for future in-depth studies [20].

This study was presented as an abstract at the 145th Annual Meeting of the American Neurological Association (ANA) (Abstract: Beriwal N, Patel RS. Mortality Risk Stratification in Pediatric Ischemic Stroke: Analysis of 4,036 Inpatients in the United States. 145th Annual Meeting of the American Neurological Association; 2020).

## Conclusions

CV risk factors like cardiomyopathies and diabetes and HEM risk factors like blood cancer, coagulation disorders, and deficiency anemias contribute significantly to in-hospital mortality risk in PAIS. The presence of CV risk factors increased the in-hospital mortality risk by 613% and, HEM risk factors increased it by 336% in PAIS inpatients in our study. A stratified evidence-based understanding of risk factors or comorbidities associated with in-hospital mortality is an imperative step in the direction to improve clinical outcomes. Understanding prevalent multifactorial comorbidities and associated mortality risk are important to increase survival rates in the PAIS patient population. It is important to devise protocols facilitating early screening, identification, and subsequent effective management of associated potential risk factors in PAIS inpatients to improve their quality of life and survival rates.

## References

[1] Greenham M, Gordon A, Anderson V, Mackay MT (2016). Outcome in childhood stroke. Stroke.

[2] Ferriero DM, Fullerton HJ, Bernard TJ (2019). Management of stroke in neonates and children: a scientific statement from the American Heart Association/American Stroke Association. Stroke.

[3] deVeber G, Andrew M, Adams C (2001). Cerebral sinovenous thrombosis in children. N Engl J Med.

[4] deVeber GA, Kirton A, Booth FA (2017). Epidemiology and outcomes of arterial ischemic stroke in children: the Canadian Pediatric Ischemic Stroke Registry. Pediatr Neurol.

[5] Krishnamurthi RV, deVeber G, Feigin VL (2015). Stroke prevalence, mortality and disability-adjusted life years in children and youth aged 0-19 years: data from the global and regional burden of stroke 2013. Neuroepidemiology.

[6] Felling RJ, Sun LR, Maxwell EC, Goldenberg N, Bernard T (2017). Pediatric arterial ischemic stroke: epidemiology, risk factors, and management. Blood Cells Mol Dis.

[7] Mackay MT, Wiznitzer M, Benedict SL, Lee KJ, Deveber GA, Ganesan V; International Pediatric Stroke Study Group (2011). Arterial ischemic stroke risk factors: the International Pediatric Stroke Study. Ann Neurol.

[8] Chiang KL, Cheng CY (2018). Epidemiology, risk factors and characteristics of pediatric stroke: a nationwide population-based study. QJM.

[9] Lo W, Stephens J, Fernandez S (2009). Pediatric stroke in the United States and the impact of risk factors. J Child Neurol.

[10] (2020). Overview of the national (nationwide) inpatient sample. https://www.hcup-us.ahrq.gov/nisoverview.jsp.

[11] (2020). NIS description of data elements. https://www.hcup-us.ahrq.gov/db/nation/nis/nisdde.jsp.

[12] Golomb MR, Fullerton HJ, Nowak-Gottl U, Deveber G; International Pediatric Stroke Study Group (2009). Male predominance in childhood ischemic stroke: findings from the International Pediatric Stroke Study. Stroke.

[13] Mallick AA, Ganesan V, Kirkham FJ (2014). Childhood arterial ischaemic stroke incidence, presenting features, and risk factors: a prospective population-based study. Lancet Neurol.

[14] Goeggel Simonetti B, Cavelti A, Arnold M (2015). Long-term outcome after arterial ischemic stroke in children and young adults. Neurology.

[15] Lopez-Espejo M, Hernandez-Chavez M, Huete I (2019). Risk factors for in-hospital and follow-up mortality after childhood arterial ischemic stroke. J Neurol.

[16] Beslow LA, Dowling MM, Hassanein SMA (2018). Mortality after pediatric arterial ischemic stroke. Pediatrics.

[17] Chung B, Wong V (2004). Pediatric stroke among Hong Kong Chinese subjects. Pediatrics.

[18] Lanthier S, Carmant L, David M, Larbrisseau A, de Veber G (2000). Stroke in children: the coexistence of multiple risk factors predicts poor outcome. Neurology.

[19] Golomb MR, Garg BP, Saha C, Williams LS (2006). Accuracy and yield of ICD-9 codes for identifying children with ischemic stroke. Neurology.

[20] Desai R, Patel U, Rupareliya C (2017). Impact of cocaine use on acute ischemic stroke patients: insights from nationwide inpatient sample in the United States. Cureus.

